# Biochar loading modulates pore accessibility and CO_2_ adsorption in dual-phase MIL-100(Al)/MIL-110(Al) hybrids

**DOI:** 10.1039/d6ra07411a

**Published:** 2026-10-19

**Authors:** Pisit Phayutcharoenkun, Rajendran Chandran, Samson B. Akindoye, Sathiyanathan Ponnan, Anh N. Phan, Amornchai Arpornwichanop, Phuet Prasertcharoensuk

**Affiliations:** a Department of Chemical Engineering, Faculty of Engineering, Chulalongkorn University Bangkok 10330 Thailand phuet.p@chula.ac.th; b Department of Chemistry, Vel Tech Rangarajan Dr. Sagunthala R&D Institute of Science and Technology Chennai 600062 India; c School of Engineering, Newcastle University Newcastle upon Tyne NE1 7RU United Kingdom; d Center of Excellence in Process and Energy Systems Engineering, Department of Chemical Engineering, Faculty of Engineering, Chulalongkorn University Bangkok 10330 Thailand

## Abstract

MIL-100(Al)/MIL-110(Al)–biochar materials were prepared by solvothermal crystallization of aluminum trimesate phases in the presence of activated bamboo-derived biochar. BC-10 and BC-20 denote nominal precursor-basis biochar additions of 10 and 20 wt%, respectively, rather than measured final composite mass fractions. PXRD reflections assigned to MIL-100(Al) and MIL-110(Al) were retained after biochar addition. Conventional TEM revealed coexisting rod-like and faceted/polyhedral particle habits in BC-0 and their spatial association with irregular carbonaceous domains in BC-10. However, peak overlap and the absence of quantitative phase analysis in HRTEM preclude determination of phase fractions or lattice-level interfaces. Among the three investigated formulations, BC-10 gave the highest equilibrium CO_2_ uptake at 298 K and a terminal equilibrium pressure of approximately 1 bar (39.59 cm^3^ STP per g; 1.77 mmol g^−1^), compared with 1.16 and 1.37 mmol g^−1^ for BC-0 and BC-20, respectively. This trend occurred despite the lower BET area of BC-10 (496.79 m^2^ g^−1^) relative to BC-0 (1148.90 m^2^ g^−1^), demonstrating that the uptake trend cannot be explained by BET surface area alone. Instrument-integrated CO_2_-TPD contributions centred below 500 °C followed the order BC-10 > BC-20 > BC-0; the separate contribution near 560 °C was excluded because thermally evolved carbon-containing species may contribute. Nonlinear Freundlich fitting produced lower RMSE, SSE and AICc values than Langmuir fitting for all samples, supporting an energetically heterogeneous adsorption response without uniquely identifying its molecular origin. Oxygenated biochar groups, Al-containing framework environments, crystallite dispersion and pore accessibility are therefore discussed as plausible contributors rather than demonstrated mechanisms. BC-10 is identified only as the best-performing composition among the formulations tested.

## Abstract 2

BC-10 gives the highest CO_2_ uptake among three tested MIL-100(Al)/MIL-110(Al)–biochar compositions despite a lower BET area than BC-0, showing that BET area alone does not describe the adsorption trend.

## Graphical abstract

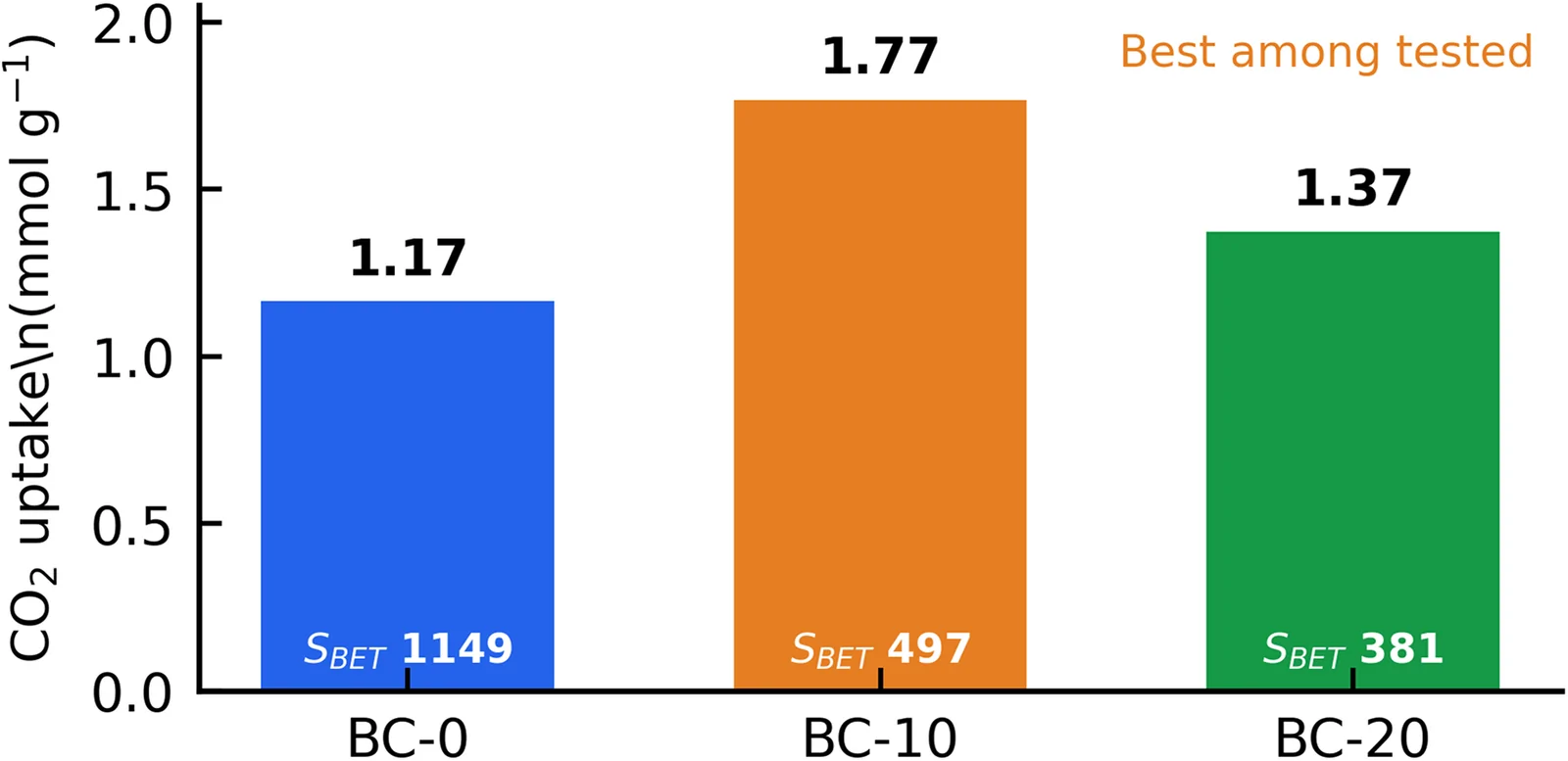

## 1. Introduction

The continuous rise in atmospheric carbon dioxide (CO_2_) concentration remains one of the most critical environmental challenges associated with anthropogenic climate change. Atmospheric CO_2_ levels have increased from the pre-industrial value of approximately 280 ppm to 422.6 ppm in 2024,^1,2^ driven primarily by fossil-fuel combustion and emissions from carbon-intensive industries, including cement, steel, and power generation.^3,4^ This persistent increase contributes substantially to global warming, climate instability, and ecological disruption. In response, carbon capture, utilization, and storage (CCUS) technologies have become essential components of global decarbonization strategies.^5^ Among available CO_2_-capture approaches, adsorption-based separation has attracted considerable attention owing to its comparatively low energy penalty, operational simplicity, and greater flexibility in regeneration relative to conventional aqueous amine scrubbing. Although amine-based technologies are commercially mature, their large-scale implementation is limited by high regeneration energy requirements, solvent degradation, equipment corrosion, and secondary environmental concerns.^5^ Accordingly, the development of robust solid adsorbents with high CO_2_ affinity, rapid adsorption kinetics, structural durability, low regeneration energy, and long-term cyclic stability remains a central objective in carbon-capture research. Among these requirements, simultaneously optimizing pore accessibility and the distribution of energetically favorable CO_2_ adsorption sites remains a major challenge for porous adsorbent design.

Metal–organic frameworks (MOFs) are among the most promising classes of porous solid adsorbents because of their high specific surface areas, tunable pore architectures, and chemically adjustable internal surfaces.^6^ Aluminum-based MOFs have received increasing attention for gas adsorption and separation owing to their relatively high thermal and hydrothermal stability, low metal toxicity, and strong metal–oxygen coordination environments.^7–10^ Among them, MIL-100(Al) and MIL-110(Al), both constructed from aluminum nodes and trimesate-based linkers, are attractive candidates because they possess distinct pore environments and complementary structural features. MIL-100(Al) contains large mesoporous cages accessible through microporous windows, whereas MIL-110(Al) exhibits one-dimensional channel-type porosity. These structural characteristics can provide diverse adsorption domains and diffusion pathways for gas molecules. Nevertheless, the practical application of pristine MOFs remains constrained by several limitations, including high synthesis costs, limited mechanical robustness, crystallite agglomeration, diffusion resistance in bulk powder form, and challenges associated with shaping and scale-up.^8,11^ These limitations have motivated the development of MOF-based hybrid materials in which crystalline frameworks are integrated with low-cost, sustainable, and structurally complementary supports.^12^

Biochar has emerged as an attractive carbonaceous support for porous adsorbents because its pore structure and oxygen-containing surface groups can influence adsorbate-accessible texture and local polarity. When a MOF is crystallized in the presence of biochar, the carbon surface may alter precursor distribution, nucleation environments and crystallite aggregation, and wider pores or interparticle voids may provide access pathways to narrower adsorption domains.^13–17^ These possibilities are general design hypotheses. Faster molecular transport, reduced diffusion resistance and improved operational stability in the present materials cannot be concluded without time-resolved uptake, breakthrough, cycling and humid-gas measurements.

The combination of an aluminum trimesate framework with activated biochar may alter crystallite distribution, pore connectivity and the chemical heterogeneity of the accessible surface. Oxygen-containing carbon functionalities can create polar adsorption environments, while coordinatively unsaturated or polar Al-containing sites may interact with CO_2_. Such interactions are chemically plausible because the quadrupole of CO_2_ responds to local electrostatic fields; nevertheless, their occurrence in the present composites is not directly demonstrated by FTIR, equilibrium adsorption or CO_2_-TPD. Previous studies of MIL-100 frameworks and functionalized carbon materials show that local metal coordination, ultramicropore confinement and oxygen-containing groups can influence CO_2_ adsorption.^18,19^ When MOFs are grown *in situ* on a biochar matrix, the carbon scaffold can provide heterogeneous nucleation sites, suppress excessive crystal agglomeration, and improve the dispersion of MOF crystallites across a porous support. Studies of Cu-BTC/aminated graphite oxide composites demonstrate that combining a MOF with a chemically modified carbon component can alter CO_2_ adsorption performance.^20^ The contribution of analogous interactions in the present biochar hybrids remains to be established.

Beyond improving structural stability, the interfacial chemistry between MOFs and biochar can significantly influence adsorption performance by regulating crystallite dispersion, pore connectivity, and the accessibility of coordinatively unsaturated metal sites. Simultaneously, oxygen-containing functional groups on activated biochar provide heterogeneous adsorption environments that promote favorable interactions with CO_2_ through Lewis acid–base and dipole–quadrupole interactions. Consequently, rational interfacial engineering has emerged as an effective strategy for improving adsorption efficiency without relying solely on increasing the specific surface area. Previous studies have demonstrated the potential of MOF/biochar composites for environmental adsorption and CO_2_-related applications, *e.g. in situ* grown NH_2_-MIL-101/biochar and MIL-100(Fe)/biochar systems for adsorption applications, as well as CuBTC- and Mg-MOF-based biochar composites investigated for CO_2_ adsorption and regeneration behavior.^21–25^ However, these reported systems have predominantly relied on the deposition or growth of a single MOF phase on a carbonaceous support. Consequently, the role of phase coexistence in regulating the structure and adsorption-relevant surface chemistry of MOF/biochar hybrids remains insufficiently understood. Although MIL-100(Al) and MIL-110(Al) are both aluminum trimesate frameworks, they exhibit distinct pore architectures: MIL-100(Al) provides cage-type environments, while MIL-110(Al) offers one-dimensional channel-type porosity. Their simultaneous integration within a biomass-derived carbon scaffold is therefore attractive because it may combine complementary pore geometries with biochar-derived interfacial functionality. Nevertheless, the deliberate construction of dual-phase MIL-100(Al)/MIL-110(Al)/biochar hybrids has not been systematically investigated. More importantly, the influence of biochar loading on the interfacial architecture, pore accessibility, heterogeneous adsorption-site distribution, and equilibrium CO_2_ adsorption behavior remains poorly understood. Establishing quantitative relationships between biochar loading, framework evolution, textural properties, and CO_2_ adsorption performance is therefore essential for developing sustainable aluminum-based MOF composites with rationally engineered adsorption characteristics.

In this study, dual-phase MIL-100(Al)/MIL-110(Al)/biochar hybrids were synthesized through the *in situ* solvothermal growth of aluminum trimesate frameworks on activated bamboo-derived biochar. The influence of biochar loading on crystal structure, morphology, textural properties, thermal stability, CO_2_-interaction-site distribution, and equilibrium CO_2_ adsorption behavior was systematically investigated. Particular attention was devoted to elucidating how the activated biochar scaffold regulates crystallite dispersion, accessible microporosity, heterogeneous adsorption sites, and interfacial interactions governing CO_2_ adsorption. By establishing a quantitative structure–property relationship between biochar loading and adsorption performance, this work provides a sustainable materials-design strategy for engineering dual-phase aluminum-based MOF hybrid adsorbents for carbon capture applications.

## 2. Materials and methods

### 2.1 Materials

All chemicals and reagents were purchased from commercial suppliers and used without further purification unless otherwise stated. Aluminum nitrate nonahydrate (Al(NO_3_)_3_·9H_2_O, 98%; Merck & Co., Inc., USA) was used as the aluminum precursor, while trimethyl 1,3,5-benzenetricarboxylate (Me_3_BTC, 98%; Sigma-Aldrich, USA) was used as the ester precursor of the trimesate linker for aluminum trimesate framework formation. Deionized (DI) water and nitric acid (HNO_3_, 65%; RCI Labscan, Thailand) were employed as the reaction medium and acid modulator, respectively. Bamboo-derived biochar (98%) was obtained from Thai Carbon & Graphite Co., Ltd. Potassium hydroxide (KOH, 85%; KemAus, Australia) was used as the chemical activating agent to enhance the porosity and surface functionality of the biochar scaffold.

### 2.2 Preparation methods

#### 2.2.1 Activation of bamboo-derived biochar

Raw bamboo-derived biochar was chemically activated using KOH to enhance its porous structure and surface functionality. Briefly, the biochar was impregnated with KOH at a KOH : biochar mass ratio of 1 : 2. The impregnated sample was then transferred to a tubular furnace and heated to 600 °C at a ramping rate of 5 °C min^−1^ under a continuous nitrogen flow of 20 L h^−1^. The activation temperature was maintained for 2 h to promote pore development within the carbon matrix. After cooling to room temperature under nitrogen, the activated product was thoroughly washed with deionized water until the filtrate reached neutral pH to remove residual KOH and soluble inorganic species. The washed solid was subsequently dried at 100 °C for 24 h. The resulting activated bamboo-derived biochar was denoted as A-BC.

#### 2.2.2 Preparation of biochar/MIL-100(Al)/MIL-110(Al) composites

The biochar/MIL-100(Al)/MIL-110(Al) composites, denoted as BC-*n*, where *n* represents the nominal biochar content, were synthesized through a solvothermal route designed to promote the *in situ* growth of aluminum trimesate frameworks on activated bamboo-derived biochar. In a typical synthesis, Al(NO_3_)_3_·9H_2_O, 0.230 g, 0.613 mmol, and Me_3_BTC, 0.104 g, 0.413 mmol, were dissolved in a mixed solvent containing 2.8 mL of deionized water and 0.77 mL of 1 M HNO_3_. Under acidic hydrothermal conditions, Me_3_BTC is expected to undergo *in situ* hydrolysis, generating trimesate species that coordinate with Al^3+^ centers to form MIL-100(Al)/MIL-110(Al) frameworks. Excess aluminum precursor was used to maintain sufficient Al^3+^ availability for framework nucleation, particularly because oxygen-containing functional groups on the biochar surface may partially coordinate Al^3+^ during the early stages of crystallization.

For the preparation of biochar-containing composites, activated bamboo-derived biochar (A-BC) was added to the precursor solution during the initial sonication step. The A-BC masses used for BC-10 and BC-20 were 37.1 and 83.5 mg, respectively, corresponding to nominal biochar loadings of 10% and 20%, respectively. These nominal loadings were calculated relative to the total initial solid mass, defined as the combined mass of Al(NO_3_)_3_·9H_2_O, Me_3_BTC, and A-BC. Sonication was performed for 1 h, followed by magnetic stirring for 30 min to promote homogeneous dispersion of the activated biochar scaffold and enhance contact between the biochar surface and the MOF precursor species. The resulting suspension was transferred into a 10 mL Teflon-lined stainless-steel autoclave and maintained at 210 °C for 3.5 h.^24^ After hydrothermal aging and cooling to ambient temperature, the resulting solids were recovered *via* filtration through a 50 µm nylon membrane. The product underwent multiple purification cycles using deionized water and methanol to remove residual acid, unreacted precursors, and weakly retained guest species. The purified solids were subsequently dried in an oven at 150 °C for 12 h to remove residual solvent molecules and improve pore accessibility before physicochemical characterization and CO_2_-TPD analysis. The pristine mixed-phase MIL-100(Al)/MIL-110(Al) material synthesized without biochar was denoted as BC-0 and used as the reference sample, while activated bamboo-derived biochar was denoted as A-BC and used as the biochar control. The overall synthesis pathway, including biochar activation, precursor dispersion, solvothermal MOF growth, post-treatment, and final material designation, is schematically illustrated in Fig. 1. The corresponding sample designations and compositions are summarized in Table 1. The BC-10 and BC-20 designations refer to nominal precursor-basis additions calculated from the combined initial masses of A-BC, Al(NO_3_)_3_·9H_2_O and Me3BTC; they are not experimentally determined final composite mass fractions. Specifically, nominal loading (%) = 100 × *m*_(A-BC_)/[*m*_(A-BC)_ + *m*_(Al(NO_3_)_3_·9H_2_O)_ + *m*_(Me_3_BTC)_]. Precursor conversion, washing losses and product yield prevent equating this ratio with the final composition.

**Fig. 1:**
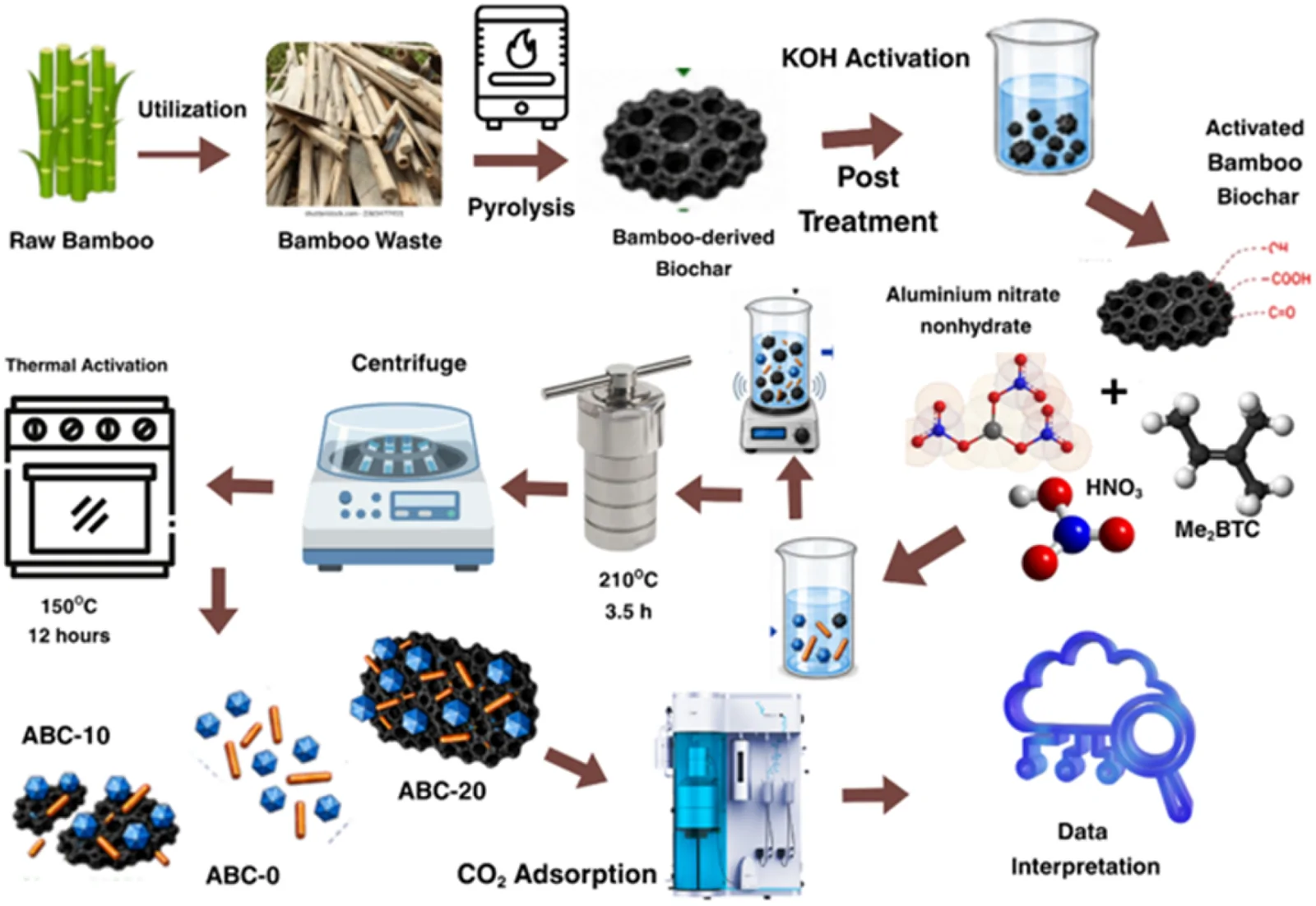
Synthesis pathway of the MIL-100(Al)/MIL-110(Al)–biochar materials.

**Table 1:** Sample designation and composition of the synthesized adsorbents

Material description	Sample code
Pristine mixed-phase MIL-100(Al)/MIL-110(Al)	BC-0
MIL-100(Al)/MIL-110(Al) composite containing 10 wt% activated bamboo-derived biochar	BC-10
MIL-100(Al)/MIL-110(Al) composite containing 20 wt% activated bamboo-derived biochar	BC-20
Activated bamboo-derived biochar	A-BC

### 2.3 Characterization

Fourier-transform infrared spectroscopy (FT-IR) was performed using a Bruker FT-IR spectrometer to identify the surface functional groups and coordination-related vibrational features of the synthesized adsorbents. Spectra were recorded over the range of 400–4000 cm^−1^ with a spectral resolution of 4 cm^−1^. The surface morphology and elemental distribution of the samples were examined using a Hitachi TM3030 scanning electron microscope, SEM, equipped with an energy-dispersive X-ray spectroscopy, EDX, detector and operated at an accelerating voltage of 15 kV. The final biochar mass fractions of BC-10 and BC-20 have not been established quantitatively. The sample labels denote nominal precursor-basis formulations; they are not confirmed by a quantitative CHON dataset reported in this study. Transmission electron microscopy (TEM) analysis was performed using a Hitachi HT7700 transmission electron microscope equipped with a LaB_6_ electron source and operated at an accelerating voltage of 120 kV. Prior to analysis, the powdered samples were ultrasonically dispersed in ethanol, and a drop of the resulting suspension was deposited onto a carbon-coated copper grid and dried at room temperature. TEM images were acquired from multiple representative regions to evaluate particle morphology, crystallite distribution, and the interfacial association of the MOF crystallites with the activated bamboo-derived biochar matrix. The crystalline structures of the adsorbents were analyzed by powder X-ray diffraction (PXRD) using a PANalytical X'Pert Pro multipurpose diffractometer equipped with an X'Celerator detector and a secondary monochromator. Measurements were conducted using Cu Kα radiation at 40 kV and 40 mA. Diffraction patterns were collected over a 2*θ* range of 5–65° with a step size of 0.03°. Thermal stability and decomposition behavior were investigated by thermogravimetric analysis (TGA) using a PerkinElmer Pyris 1. Each sample was heated from 25 to 800 °C at 10 °C min^−1^ under a nitrogen flow of 20 mL min^−1^ to evaluate solvent removal, thermal degradation, decomposition behavior, and residual solid content. The textural properties of the samples were determined from nitrogen adsorption–desorption isotherms measured at 77 K using a Thermo Scientific surfer gas adsorption porosimeter. Nitrogen adsorption–desorption isotherms were collected over a relative pressure (*P*/*P*_0_) range of 0.01–1.0. The specific surface area was calculated using the Brunauer–Emmett–Teller (BET) method, and pore structure parameters were derived from the adsorption–desorption data.

### 2.4 Surface basicity analysis by CO_2_-TPD

CO_2_ temperature-programmed desorption (CO_2_-TPD) was used to compare CO_2_ retention under a common experimental protocol. Approximately 100 mg of sample was degassed at 200 °C for 24 h under He (30 mL min^−1^), cooled to 50 °C, exposed to 5% CO_2_/He (30 mL min^−1^) for 1 h and purged with He for 30 min. Desorption was recorded from 50 to approximately 750 °C at 10 °C min^−1^ under He using a thermal-conductivity detector. Instrument-derived integrated amounts were resolved using Gaussian components in OriginPro. The integration windows and fitted components are operational descriptors rather than unique chemical assignments. Because peak areas depend on calibration, adsorption stoichiometry, heating rate, baseline treatment and desorption kinetics, the values are reported as comparative CO_2_ desorption amounts under the applied TPD conditions, not as absolute adsorption-site densities. The contribution centred near 560 °C was excluded from the lower-temperature comparison because framework or biochar decomposition may release carbon-containing species in this region.

## 3. Results and discussion

### 3.1 Phase evolution and crystallographic analysis

Powder X-ray diffraction (PXRD) was employed to examine the crystalline structure and phase evolution of activated bamboo-derived biochar (A-BC), the pristine mixed-phase MOF (BC-0), and the corresponding biochar-containing hybrids (BC-10 and BC-20) (Fig. 2A). As shown in Fig. 2A(a), A-BC exhibited broad diffraction features centered at approximately 2*θ* = 20–30° and 42–45°, which can be assigned to the partially ordered carbonaceous domains associated with the (002) and (100)/(101) reflections of turbostratic carbon, respectively.^25^ The broad, low-intensity nature of these features indicates that the activated biochar exhibits predominantly amorphous carbon characteristics with limited graphitic ordering. The absence of intense crystalline reflections further suggests that no major crystalline inorganic phases are present within the detection limit of PXRD, although minor mineral residues originating from the biomass precursor cannot be completely excluded.

**Fig. 2:**
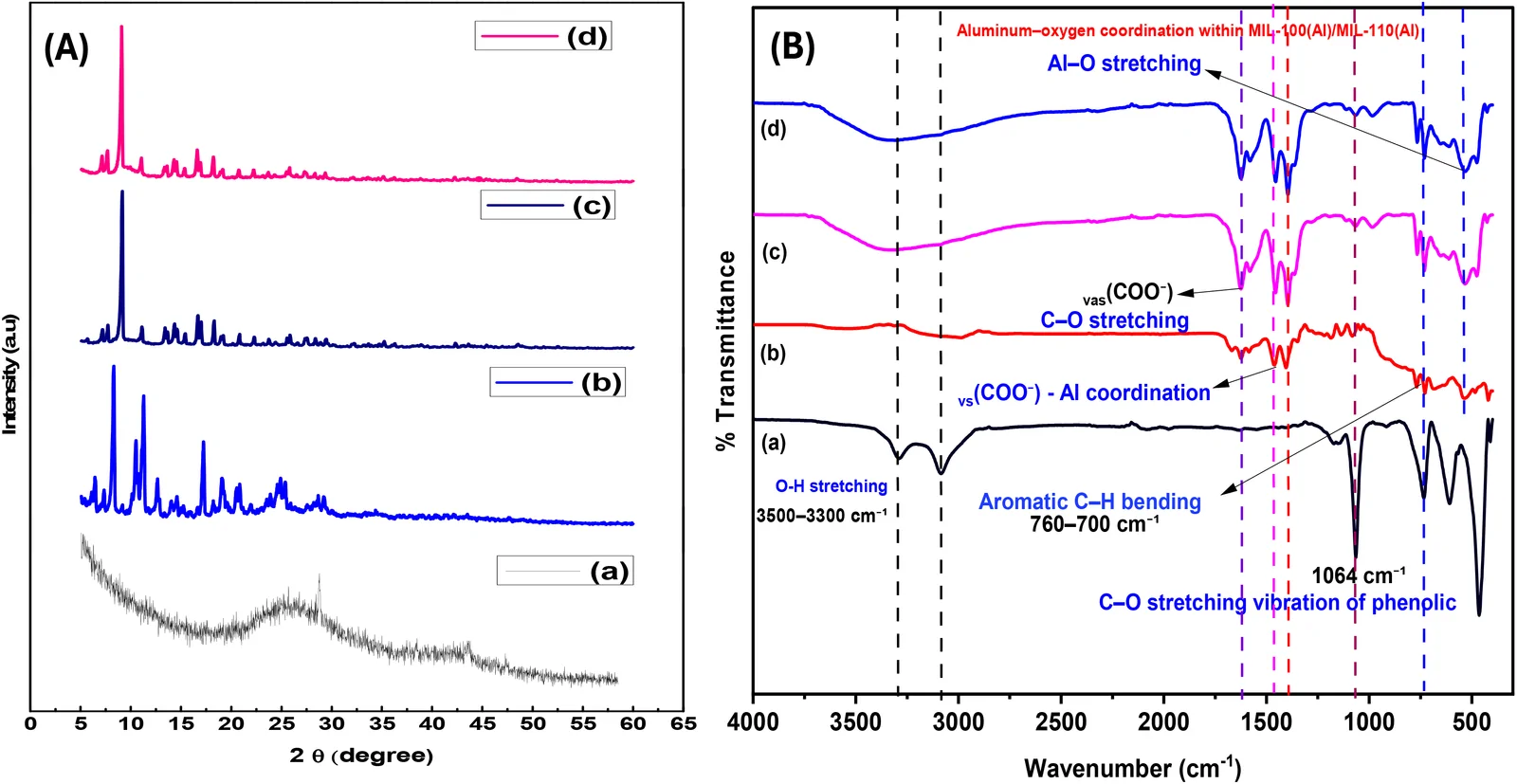
(A) PXRD patterns of (a) A-BC, (b) BC-0, (c) BC-10, and (d) BC-20. (B) FTIR spectra of (a) A-BC, (b) BC-0, (c) BC-10, and (d) BC-20.

The PXRD pattern of BC-0 contains several well-defined reflections in the low-angle region that are consistent with the formation of aluminum trimesate frameworks. Reflections at approximately 2*θ* = 5.94°, 6.68°, and 11.14° are assigned to characteristic features reported for MIL-100(Al), whereas reflections near 9.56°, 13.40°, and 20.55° are consistent with MIL-110(Al).^24,26–28^ The simultaneous observation of these two sets of reflections indicates the coexistence of MIL-100(Al)-like and MIL-110(Al)-like crystalline domains under the applied solvothermal conditions. However, several reflections of aluminum trimesate frameworks may partially overlap, particularly in the low-angle region. Their positions and relative intensities may also be affected by hydration, residual guest molecules, preferred orientation, crystallite size, and instrumental broadening. The diffraction data are therefore interpreted as evidence consistent with phase coexistence rather than definitive proof of a crystallographically integrated dual-phase framework.

BC-10 and BC-20 retain the principal reflections assigned to both aluminum trimesate phases, although the reflections become progressively weaker and broader with increasing nominal biochar addition. This change may reflect dilution by the amorphous carbonaceous component, attenuation of X-ray scattering, and/or a decrease in coherent crystallite size. Because quantitative phase refinement and nanoscale crystallographic imaging were not performed, the relative fractions of MIL-100(Al) and MIL-110(Al) cannot be determined, and PXRD cannot distinguish between physically coexisting phases, simultaneous crystallization on the biochar surface, and direct interfacial integration. Upon incorporation of 10 wt% activated biochar, the BC-10 hybrid, Fig. 2A(c), retained the principal reflections associated with both MIL-100(Al) and MIL-110(Al), indicating that the dual-phase framework structure was largely preserved after integration with the carbon scaffold. However, the diffraction peaks became less intense and slightly broader than those of BC-0. This reduction in peak intensity can be attributed to the dilution effect introduced by the amorphous biochar phase, attenuation of X-ray scattering by the carbonaceous matrix, and a possible decrease in the coherent crystalline domain size of the MOF component. The absence of additional intense reflections suggests that no major crystalline impurity phases were generated during composite formation within the detection limit of PXRD. For the BC-20 hybrid, Fig. 2A(d), the characteristic reflections of both MIL-100(Al) and MIL-110(Al) remained discernible, although their intensities were further reduced and the background contribution became more pronounced. This behavior is consistent with the higher fraction of amorphous biochar in the composite. Activated biochar commonly exhibits a broad diffuse halo centered around 2*θ* = 20–25°, characteristic of turbostratic carbon with limited long-range ordering.^29,30^ In BC-20, this amorphous carbon contribution overlaps with the background of the MOF diffraction pattern, leading to generalized baseline elevation rather than a distinct biochar reflection. Importantly, the persistence of the phase-specific reflections of both MIL-100(Al) and MIL-110(Al) even at 20 wt% biochar loading demonstrates that the dual-phase aluminum trimesate framework is retained after incorporation into the biochar scaffold. Collectively, these results indicate that the *in situ* solvothermal growth strategy enables the formation of dual-phase MIL-100(Al)/MIL-110(Al)/biochar hybrids without substantial disruption of the primary crystalline framework. Biochar incorporation is associated with changes in particle distribution and aggregation. Its surface may influence precursor dispersion or provide heterogeneous nucleation environments, although a crystallization-directing role has not been established by experimentation.

Fourier-transform infrared spectroscopy (FTIR) was used to evaluate the functional groups, coordination environment, and possible interfacial interactions in A-BC, BC-0, BC-10, and BC-20, as shown in Fig. 2B. The A-BC spectrum exhibits a broad absorption band in the region of 3500–3300 cm^−1^, which is assigned to O–H stretching vibrations associated with hydroxyl, phenolic, and carboxylic surface groups on the activated biochar. The band observed at approximately 1084 cm^−1^ can be attributed to C–O stretching vibrations, indicating the presence of oxygen-containing carbonaceous functionalities remaining after carbonization and KOH activation. In addition, the absorption feature near 752 cm^−1^ is associated with aromatic C–H out-of-plane bending, consistent with partially ordered aromatic domains in the turbostratic carbon matrix. For the pristine mixed-phase MOF, BC-0, the broad absorption envelope around 3500–3300 cm^−1^ is attributed to O–H stretching vibrations from terminal hydroxyl groups associated with aluminum-oxo clusters and adsorbed or confined water molecules within the porous framework.^30–32^ The characteristic carboxylate vibrations of coordinated trimesate linkers are clearly observed, with bands at approximately 1625 and 1445 cm^−1^ corresponding to the asymmetric, *ν*_as_(COO^−^), and symmetric, *ν*_s_(COO^−^), stretching modes of coordinated carboxylate groups, respectively.^31,33^ The separation between these bands, Δ*ν* = *ν*_as_(COO^−^) − *ν*_s_(COO^−^), is approximately 180 cm^−1^, which is below 200 cm^−1^ and suggests that the carboxylate groups are predominantly involved in bridging coordination with aluminum centers within the MIL-100(Al)/MIL-110(Al) framework.

After incorporation of activated biochar, the FTIR spectra of BC-10 and BC-20 retain the principal carboxylate stretching bands of the aluminum trimesate framework, indicating that the primary coordination environment of the MIL-100(Al)/MIL-110(Al) phases is largely preserved. The persistence of these framework-related vibrations is consistent with the PXRD results, which show that the characteristic crystalline features of the dual-phase MOF are retained after biochar incorporation. Compared with BC-0, the biochar-containing hybrids exhibit broader O–H absorption in the 3500–3300 cm^−1^ region, particularly for BC-20. This broadening can be attributed to the increased contribution of hydroxyl- and carboxyl-containing groups from the activated biochar, together with possible hydrogen-bonding interactions between the biochar surface and the aluminum trimesate framework.^34–36^ The relative intensities of the carboxylate-related bands decrease with increasing biochar loading, which is consistent with the increasing contribution of the carbonaceous phase and the corresponding dilution of the MOF vibrational signal. No additional intense absorption bands attributable to crystalline secondary by-products are observed, suggesting that biochar incorporation does not substantially alter the fundamental aluminum–carboxylate coordination environment within the detection limits of FTIR. These FTIR results support the coexistence of the activated biochar scaffold and the aluminum trimesate framework, while suggesting possible non-covalent interfacial interactions, particularly hydrogen bonding, within the hybrid structure.^36^ The absence of significant band shifts further indicates that the interaction between the MOF framework and biochar is predominantly interfacial rather than arising from the formation of new covalent bonds.

### 3.2 Morphological evolution of A-BC and BC-*n* hybrids

Scanning electron microscopy, SEM, was used to investigate the morphological evolution of the activated bamboo-derived biochar scaffold, A-BC, the pristine mixed-phase MOF, BC-0, and the corresponding biochar-containing hybrids, BC-10 and BC-20, as shown in Fig. 3.

**Fig. 3:**
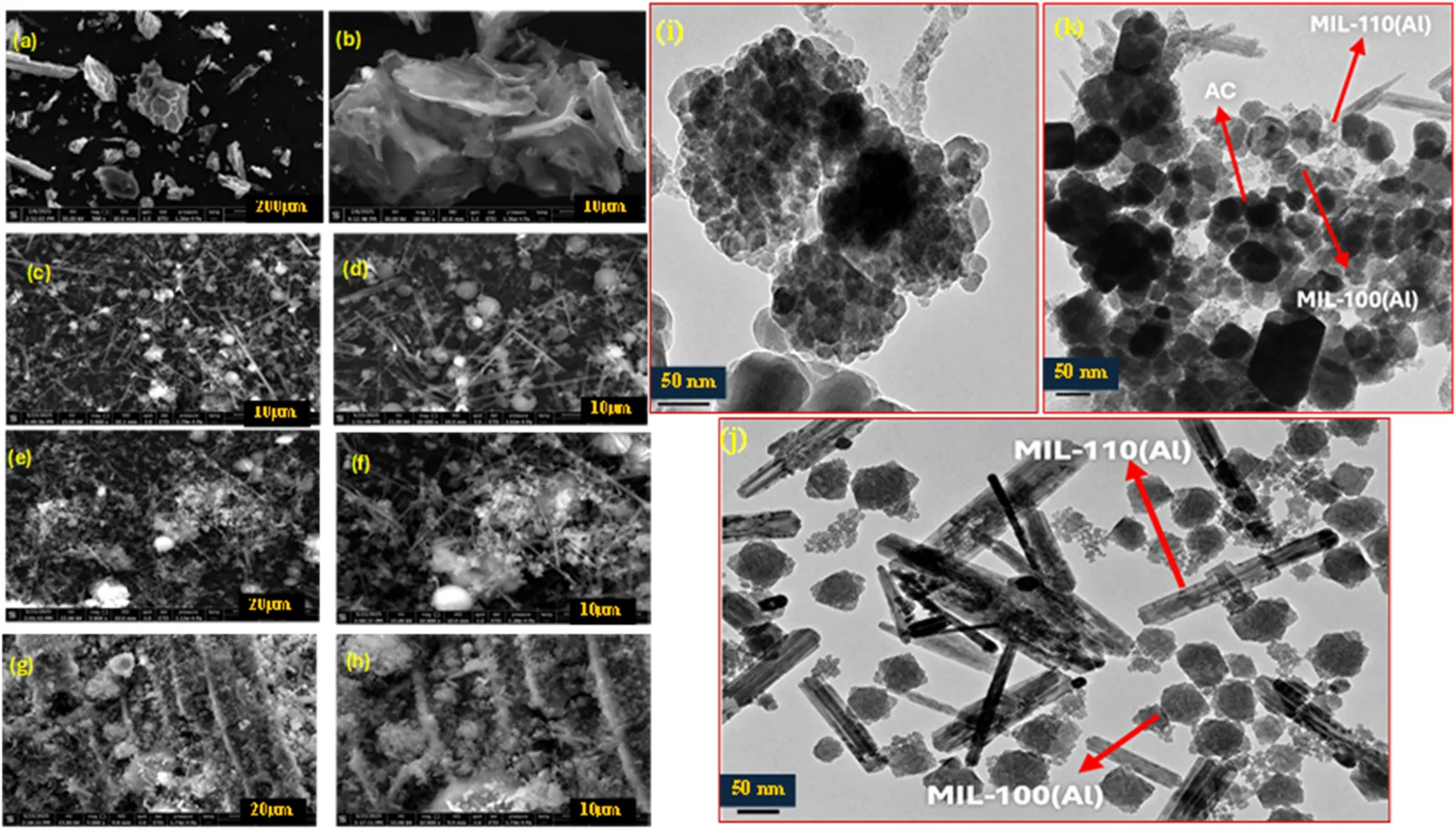
SEM micrographs of (a and b) A-BC, (c and d) BC-0, (e and f) BC-10 and (g and h) BC-20, together with conventional TEM images of (i) A-BC, (j) BC-0 and (k) BC-10. All TEM scale bars are 50 nm.

The A-BC sample (Fig. 3a and b) exhibits an irregular, fragmented carbonaceous morphology with a rough surface and visible open cavities. This morphology is consistent with the structural disruption and pore development typically produced during carbonization followed by KOH chemical activation of lignocellulosic biomass.^36^ The presence of surface roughness and accessible voids suggests that A-BC can provide a heterogeneous support surface for precursor adsorption and subsequent MOF nucleation during solvothermal synthesis. The pristine mixed-phase MOF, BC-0 (Fig. 3c and d), displays a heterogeneous assembly of crystalline particles, including elongated rod-like structures and rounded or polyhedral crystallites. These morphologies are consistent with the coexistence of MIL-110(Al)-type channel structures and MIL-100(Al)-type cage-based crystallites, respectively, as suggested by the PXRD analysis. The particles in BC-0 appear partially agglomerated, forming dense intergrown domains with relatively limited interparticle spacing. Such agglomeration is commonly observed in solvothermally synthesized MOF powders, particularly under conditions where rapid nucleation and crystal growth occur in the absence of an external support matrix.^37–41^ After incorporation of 10 wt% activated biochar, the BC-10 hybrid, Fig. 3e and f, shows a more dispersed arrangement of MOF crystallites distributed over the carbonaceous scaffold. For each sample, the left and right images were acquired at magnifications of 5000× and 100 00×, respectively. Conventional TEM provides complementary nanoscale morphology evidence (Fig. 3i–k). A-BC appears as irregular, electron-dense aggregates assembled from smaller carbonaceous domains, without the well-defined rod-like or faceted habits observed for BC-0. BC-0 contains elongated rod-like crystallites together with rounded faceted/polyhedral particles. Rod-like MIL-110(Al) and faceted or octahedral MIL-100(Al) morphologies have been reported for phase-selective aluminum trimesate syntheses,^38,39^ so the coexistence of these contrasting habits is morphologically consistent with the PXRD-based phase assignment. Particle shape is not phase-specific, however, and cannot replace diffraction. In BC-10, rod-like and faceted particles occur in close spatial association with irregular carbon-rich aggregates, supporting framework crystallization in the presence of biochar and altered particle dispersion. Compared with BC-0, the crystalline domains appear less densely packed, and smaller MOF particles and elongated crystallites partially decorate the biochar surface. This morphological change is consistent with activated biochar acting as a heterogeneous nucleation support, in which oxygen-containing surface functionalities may facilitate local Al^3+^ coordination and promote spatially distributed framework growth.^42,43^ The resulting hybrid architecture may improve accessibility to external and interparticle regions by reducing the extent of MOF crystallite aggregation.

For the BC-20 hybrid (Fig. 3g and h), the carbonaceous phase becomes more prominent, and the MOF crystallites appear more extensively embedded within the biochar-rich matrix. The surface is covered by a dense population of small particles and rod-like crystallites, indicating that higher biochar loading provides additional nucleation surfaces for MOF growth. However, the increased carbon fraction also appears to mask or surround the crystalline MOF domains partially. This observation is consistent with PXRD and textural-property trends, in which increased biochar loading attenuates MOF diffraction intensity and may reduce accessible surface area. Therefore, while biochar incorporation promotes crystallite dispersion, excessive biochar loading may lead to partial coverage or confinement of the MOF phase within the carbon scaffold. These SEM observations indicate that biochar incorporation regulates MOF crystallite dispersion in a loading-dependent manner. Moderate biochar loading, as observed for BC-10, reduces crystallite agglomeration and promotes a more open hybrid architecture. In contrast, excessive loading, as observed for BC-20, leads to a biochar-dominant morphology that may partially restrict access to MOF domains. Such morphological evolution is expected to improve the accessibility of adsorption sites by minimizing particle agglomeration and facilitating gas diffusion throughout the hybrid architecture.

### 3.3 Textural properties and pore structure

The textural properties and pore architecture of activated bamboo-derived biochar (A-BC), the pristine mixed-phase aluminum trimesate framework (BC-0), and the biochar-containing hybrids (BC-10 and BC-20) were evaluated by N_2_ adsorption–desorption analysis at 77 K. The corresponding isotherms are presented in Fig. 4, and the derived textural parameters are summarized in Table 2. All samples exhibited a pronounced N_2_ uptake at low relative pressure, particularly below *P*/*P*_0_ < 0.05, indicating rapid micropore filling and the presence of abundant microporous domains. This low-pressure uptake is characteristic of type I behavior, whereas the adsorption–desorption hysteresis and additional uptake at intermediate-to-high relative pressure indicate a contribution from wider accessible pore domains, giving the overall profiles mixed type I/type IV characteristics. The further rise near *P*/*P*_0_ ≈ 1.0 suggests additional contributions from wider pore domains and/or interparticle textural voids at higher relative pressure. Among the investigated materials, BC-0 displayed the highest overall N_2_ uptake, consistent with the most highly developed accessible pore network of the pristine mixed-phase framework. The progressive decrease in N_2_ uptake from BC-0 to BC-10 and BC-20 reflects the loading-dependent reduction in accessible surface area and pore volume following biochar incorporation; nevertheless, the retention of steep low-pressure uptake in both hybrid samples confirms that substantial microporosity is preserved after integration of the activated carbon scaffold.

**Fig. 4:**
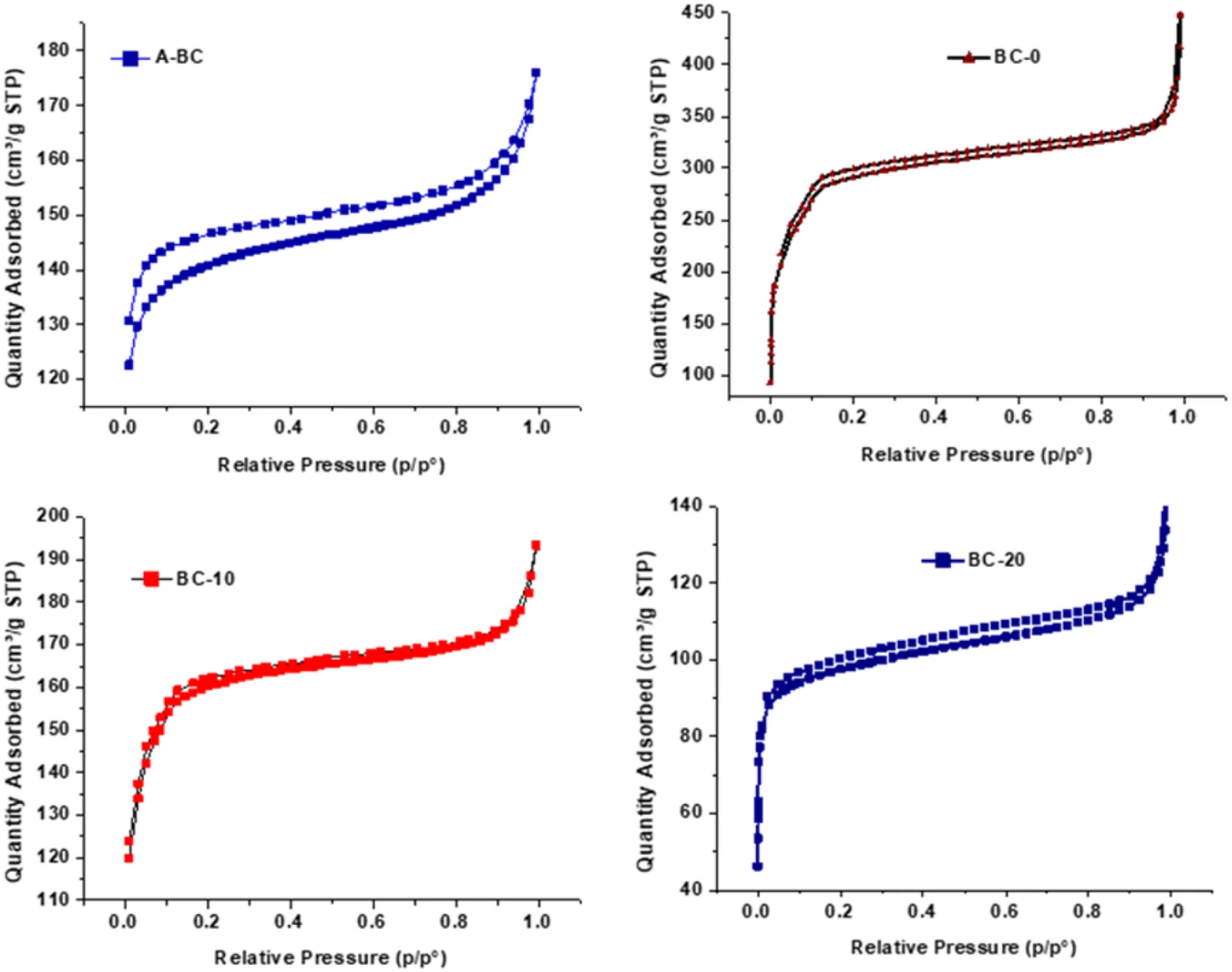
N_2_ adsorption–desorption isotherms of A-BC, BC-0, BC-10, and BC-20.

**Table 2:** Textural properties of A-BC, BC-0, BC-10, and BC-20 derived from N_2_ adsorption–desorption analysis at 77 K

Sample	*S*_BET_ (m^2^ g^−1^)	*S*_micro_ (m^2^ g^−1^)	*S*_Ext_ (m^2^ g^−1^)	*D*_avg_ (Å)	*V*_pore_ (cm^3^ g^−1^)
A-BC	432.10	355.20	76.90	23.23	0.251
BC-0	1148.90	981.50	167.30	22.22	0.694
BC-10	496.79	372.99	123.80	22.08	0.274
BC-20	381.23	326.60	54.65	24.27	0.2314

A-BC exhibited a BET specific surface area (*S*_BET_) of 432.1 m^2^ g^−1^ and a total pore volume (*V*_pore_) of 0.251 cm^3^ g^−1^. The *t*-plot analysis yielded a micropore surface area (*S*_micro_) of 355.2 m^2^ g^−1^ and a micropore volume (*V*_micro_) of 0.184 cm^3^ g^−1^, confirming the predominantly microporous nature of the activated biochar. This interpretation is consistent with the A-BC isotherm, in which the sharp uptake at low *P*/*P*_0_ reflects rapid filling of narrow micropores generated during chemical activation, while the limited but discernible uptake at higher relative pressure indicates the presence of additional wider pore domains or interparticle voids. Such pore development is typical of lignocellulosic biochar after KOH activation, where chemical etching, carbon matrix rearrangement, and partial gasification promote the formation of narrow pores within the carbonaceous framework.^44^

Among all samples, BC-0 exhibited the most highly developed porous structure, with the highest *S*_BET_ of 1148.9 m^2^ g^−1^, *S*_micro_ of 981.5 m^2^ g^−1^, and *V*_pore_ of 0.694 cm^3^ g^−1^. The high *S*_micro_/*S*_BET_ ratio of approximately 85.4%, together with the pronounced low-pressure N_2_ uptake, confirms the strongly micropore-dominant character of the pristine framework. This highly accessible pore network is consistent with the coexistence of MIL-100(Al) and MIL-110(Al) phases identified by PXRD, in which cage-type and channel-type pore environments collectively contribute to the overall porosity of the mixed-phase aluminum trimesate structure. Incorporation of 10 wt% activated biochar decreased *S*_BET_ to 496.79 m^2^ g^−1^, *S*_micro_ to 372.99 m^2^ g^−1^, and *V*_pore_ to 0.274 cm^3^ g^−1^ for BC-10. The lower adsorption capacity of BC-10 relative to BC-0 throughout the N_2_ isotherm is consistent with the reduced accessible surface area and pore volume. This decrease can be attributed to dilution of the high-surface-area MOF phase by the carbon scaffold, as well as to partial coverage or restriction of the framework micropores during hybrid formation. Nevertheless, BC-10 retained substantial microporosity, as evidenced by its steep low-pressure uptake and *S*_micro_/*S*_BET_ ratio of approximately 75.1%. Importantly, although the absolute surface area decreased after biochar incorporation, the relative non-microporous surface contribution increased from approximately 14.6% for BC-0 to 24.9% for BC-10, indicating that moderate biochar addition promotes a more balanced accessible pore structure (Table 2). This redistribution of porosity may be advantageous for molecular transport by preserving micropore-derived adsorption sites while increasing the contribution of wider accessible pore domains within the hybrid architecture.^45–47^

Further increasing the biochar content to 20 wt% resulted in an additional decline in *S*_BET_, *S*_micro_, and *V*_pore_ to 381.2 m^2^ g^−1^, 326.6 m^2^ g^−1^, and 0.231 cm^3^ g^−1^, respectively. Consistent with these values, BC-20 exhibited lower N_2_ uptake than BC-0 and BC-10 across most of the relative-pressure range. Although BC-20 remained predominantly microporous, with an *S*_micro_/*S*_BET_ ratio of approximately 85.7%, its markedly reduced total surface area, pore volume, and non-microporous surface contribution indicate that excessive biochar incorporation decreases the relative contribution of the highly porous MOF phase and may partially restrict access to framework-derived pores and it was confirmed in the Table 2. This behavior is consistent with a crystalline dilution effect, in which increasing the carbon fraction lowers the relative abundance of high-surface-area MOF domains and diminishes overall pore accessibility.^48^ Collectively, the N_2_ adsorption–desorption isotherms and textural parameters demonstrate that biochar loading strongly governs the accessible pore architecture of the MIL-100(Al)/MIL-110(Al)-based hybrids. BC-0 provides the highest intrinsic surface area, microporosity, and total pore volume, reflecting the dominant contribution of the mixed-phase MOF framework. By contrast, BC-10 offers the most favorable textural balance among the hybrid samples, retaining substantial microporous surface area while exhibiting the highest relative non-microporous surface contribution, which is expected to improve accessibility and molecular transport within the composite structure. Excessive biochar loading in BC-20, however, reduces the accessible surface area and pore volume, indicating partial masking or restriction of MOF-derived porosity by the carbonaceous matrix. These results identify a nominal biochar loading of 10 wt% as the most favorable among the investigated formulations for achieving a balanced accessible pore architecture.

### 3.4 Thermal decomposition behavior under N_2_

Thermogravimetric analysis (TGA) was conducted to evaluate the thermal behaviour and decomposition characteristics of A-BC, BC-0, BC-10, and BC-20, as shown in Fig. 5. The measurements were performed under a nitrogen atmosphere from ambient temperature to 800 °C at a heating rate of 10 °C min^−1^. The pristine mixed-phase MOF, BC-0, exhibited a multistep mass-loss profile characteristic of aluminum-based metal–organic frameworks.

**Fig. 5:**
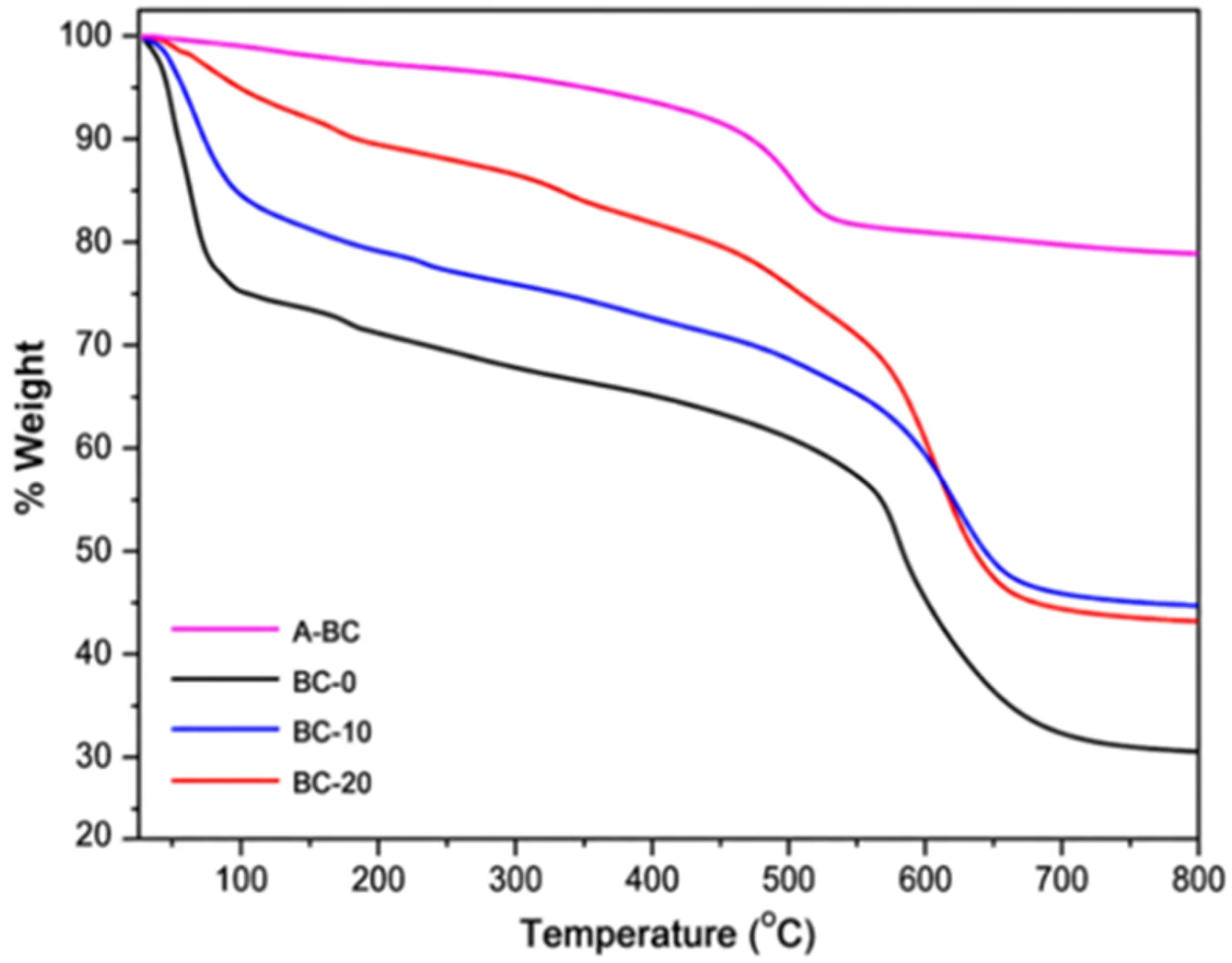
Thermogravimetric analysis, TGA, profiles of A-BC, BC-0, BC-10, and BC-20.

An initial mass loss of approximately 25 wt% below 120 °C is attributed to the removal of physically adsorbed water and residual solvent molecules retained within the porous framework, consistent with the hydrophilic nature of Al–O clusters and the presence of accessible internal pores.^49,50^ A further gradual mass loss between approximately 120 and 480 °C can be assigned to the release of more strongly retained guest molecules, coordinated water, and progressive thermal destabilization of the organic trimesate linkers. The major mass-loss event between approximately 480 and 650 °C corresponds to decomposition of the organic linker and collapse of the MIL-100(Al)/MIL-110(Al) framework, leaving a residual mass of approximately 30 wt% at 800 °C, most likely associated with aluminum-containing inorganic residues such as Al_2_O_3_.^51^ In contrast, A-BC exhibited substantially lower overall mass loss throughout the investigated temperature range, with only a minor decrease of approximately 2–3 wt% below 150 °C and a high residual mass of approximately 79 wt% at 800 °C. The gradual weight decrease observed between approximately 200 and 500 °C is attributed to the decomposition of residual oxygen-containing surface groups, including hydroxyl, carboxyl, carbonyl, and phenolic moieties, through dehydration, decarboxylation, and dehydroxylation processes.^52^ The high final residue reflects the carbon-rich and thermally stable nature of the activated bamboo-derived biochar scaffold.

The biochar-containing hybrids exhibited intermediate thermal behavior between BC-0 and A-BC. BC-10 retained approximately 47 wt% of its initial mass at 800 °C, while BC-20 retained approximately 43 wt%, both markedly higher than BC-0. Relative to the pristine framework, the hybrids showed less pronounced low-temperature mass loss, indicating a reduced relative contribution of physisorbed guest species after biochar incorporation. Their principal decomposition regions remained broadly comparable to that of BC-0, suggesting that the characteristic thermal degradation behavior of the aluminum trimesate framework was largely preserved after hybrid formation.^53^ The increased residual masses of BC-10 and BC-20 relative to BC-0 are therefore attributed to the combined contribution of the thermally stable carbonaceous scaffold and the aluminum-containing inorganic residue formed after framework decomposition.^54,55^

These measurements characterize decomposition under N_2_ and do not establish framework preservation, hydrothermal resistance, humid-gas stability or cyclic adsorption–desorption durability.

### 3.5 Surface basicity and CO_2_-interaction site distribution by CO_2_-TPD

CO_2_ temperature-programmed desorption, CO_2_-TPD, was performed to evaluate the distribution and relative strength of CO_2_-interacting basic sites in BC-0, BC-10, and BC-20. The desorption profiles recorded between 50 and 500 °C are shown in Fig. 6. This temperature range covers weakly retained CO_2_ species at low temperatures as well as more strongly retained CO_2_-containing species at elevated temperatures, while minimizing overlap with the major framework decomposition region identified by TGA. All samples exhibited broad and partially overlapping desorption features, indicating the presence of heterogeneous CO_2_-interaction sites. In the low-temperature region, below approximately 150 °C, the desorption signals are attributed mainly to weakly adsorbed CO_2_ species associated with physisorption, surface hydroxyl groups, and weak polar interactions.^55,56^ The intermediate-temperature region, approximately 150–280 °C, corresponds to medium-strength sites, which may involve stronger interactions with oxygen-containing surface groups and coordinatively unsaturated or polar aluminum sites within the MIL-100(Al)/MIL-110(Al) framework.^57,58^ Compared with BC-0, the BC-10 hybrid shows a more intense desorption response across the medium- and strong-interaction regions. In particular, the desorption feature centered at approximately 331 °C suggests an increased contribution from stronger CO_2_-retention sites after incorporation of 10 wt% activated biochar. This enhancement is consistent with the contribution of additional oxygen-containing functionalities from the biochar scaffold, which may promote stronger CO_2_–surface interactions through polar, acid–base, and quadrupole-associated effects.^59–61^ However, the high-temperature feature near approximately 464 °C should be interpreted cautiously because it may include contributions from strongly retained CO_2_-containing species as well as thermally released carbon-containing groups from the framework or biochar surface. Therefore, the reported CO_2_-TPD-derived basicity values should be regarded as comparative site-density indicators under the applied TPD conditions rather than absolute equilibrium CO_2_ adsorption capacities.

**Fig. 6:**
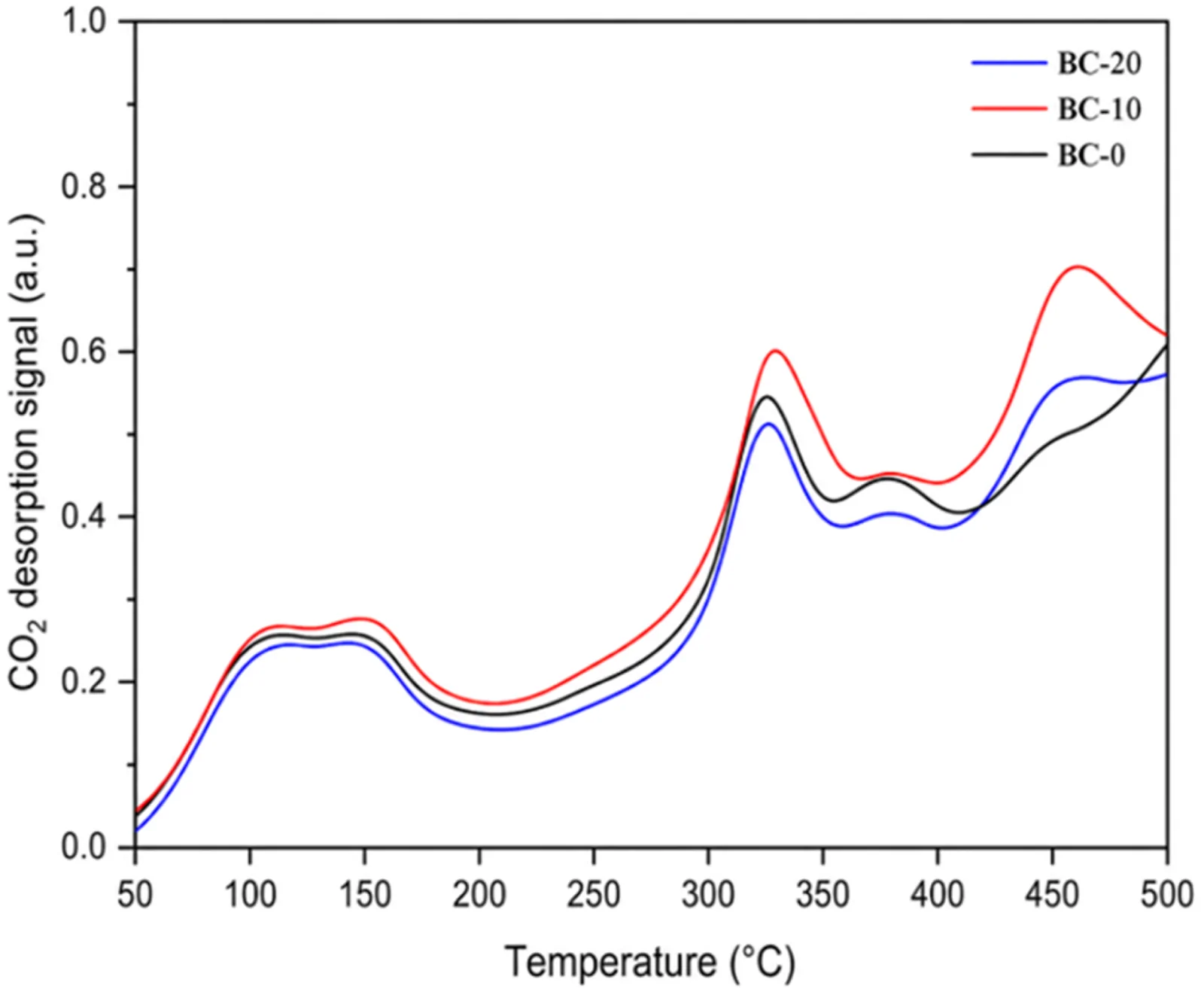
CO_2_ temperature-programmed desorption, CO_2_-TPD, profiles of BC-0, BC-10, and BC-20.

Quantitative analysis was performed by deconvoluting the CO_2_-TPD profiles into weak, medium, strong, and very strong interaction regimes, as summarized in Table 3. Compared with BC-0, BC-10 exhibited higher contributions across the resolved weak, medium, and strong interaction regimes, including a strong-site density of 2.781 mmol g^−1^ that was not clearly resolved for the pristine mixed-phase framework. The full deconvoluted profiles yielded total CO_2_-TPD-derived basicities of 11.538, 16.542, and 13.256 mmol g^−1^ for BC-0, BC-10, and BC-20, respectively. Although these total values provide a complete overview of the desorption profiles, they should be interpreted cautiously because the very strong 400–500 °C region may partly overlap with thermally released carbon-containing species.

**Table 3:** Quantitative distribution of CO_2_-TPD-derived basic sites over BC-0, BC-10, and BC-20

Sample	Weak sites (50–150 °C) (mmol g^−1^)	Medium sites (150–280 °C) (mmol g^−1^)	Strong sites (280–400 °C) (mmol g^−1^)	Very strong site (400–500 °C) (mmol g^−1^)	Total CO_2_-TPD-derived basicity (mmol g^−1^)
BC-0	0.8219	1.7462	n.d.	8.9706	11.538
BC-10	1.2872	2.7583	2.7811	9.7153	16.54
BC-20	1.0430	2.0180	1.9227	8.2724	13.25

In contrast to BC-10, BC-20 exhibited lower contributions across the resolved weak-to-strong interaction regimes despite its higher biochar loading. This decrease is consistent with the textural analysis, which showed a substantial reduction in BET surface area and micropore surface area at a nominal biochar loading of 20 wt%. The lower CO_2_-TPD response of BC-20 suggests that excessive carbon incorporation may partially restrict access to MOF-derived micropores and mask active framework sites. Similar trade-offs between carbon loading, pore accessibility, and CO_2_-interaction-site availability have been reported for carbon-based and hybrid solid sorbents.^62,63^ Collectively, these results indicate that controlled biochar incorporation modulates the distribution of accessible CO_2_-interacting sites, with BC-10 exhibiting the most favorable balance between accessible MOF porosity and biochar-derived surface functionality among the investigated samples. To minimize the influence of the high-temperature region, where CO_2_ desorption may overlap with thermally released carbon-containing species, the cumulative weak-to-strong site density below 400 °C was calculated from the deconvoluted CO_2_-TPD profiles, as summarized in Table 4. This below 400 °C metric was used as the more reliable comparative basis for evaluating biochar-induced changes in accessible CO_2_-interacting sites.^64–68^ BC-10 exhibited the highest cumulative weak-to-strong site density of 6.827 mmol g^−1^, corresponding to a 165.8% increase relative to BC-0. BC-20 also showed a higher cumulative weak-to-strong site density than BC-0 but remained lower than BC-10, consistent with partial loss of accessible porosity at higher biochar loading. These results further indicate that a nominal biochar loading of 10 wt% provides the most effective balance between additional CO_2_-interacting surface functionality and preservation of accessible MOF-derived porosity.

**Table 4:** Cumulative weak-to-strong CO_2_-TPD-derived basic sites, excluding the very strong 400–500 °C region

Sample	Cumulative weak-to-strong sites, 50–400 °C (mmol g^−1^)	Increase relative to BC-0 (%)	Predominant resolved site regime
BC-0	2.568	Baseline	Medium
BC-10	6.827	165.8	Medium-to-strong
BC-20	4.984	94.1	Medium-to-strong

### 3.6 Surface characteristics and CO_2_ adsorption behavior

The equilibrium CO_2_ adsorption–desorption isotherms of BC-0, BC-10, and BC-20 are presented in Fig. 7, while the corresponding adsorption characteristics are summarized in Table 5. The sharp increase in CO_2_ uptake at very low relative pressures (*P*/*P*_0_ < 0.01) indicates that adsorption is predominantly governed by micropore filling in ultra-micropores, where overlapping adsorption potentials generate strong adsorption fields for CO_2_ molecules. This behavior is characteristic of porous carbons and MOF-derived adsorbents with abundant narrow micropores.^46,47,69–71^ Among the synthesized composites, BC-10 exhibited the highest equilibrium CO_2_ adsorption capacity (39.59 cm^3^ per STP g; 1.77 mmol g^−1^), followed by BC-20 (30.77 cm^3^ STP per g; 1.37 mmol g^−1^) and BC-0 (26.11 cm^3^ STP per g; 1.16 mmol g^−1^).

**Fig. 7:**
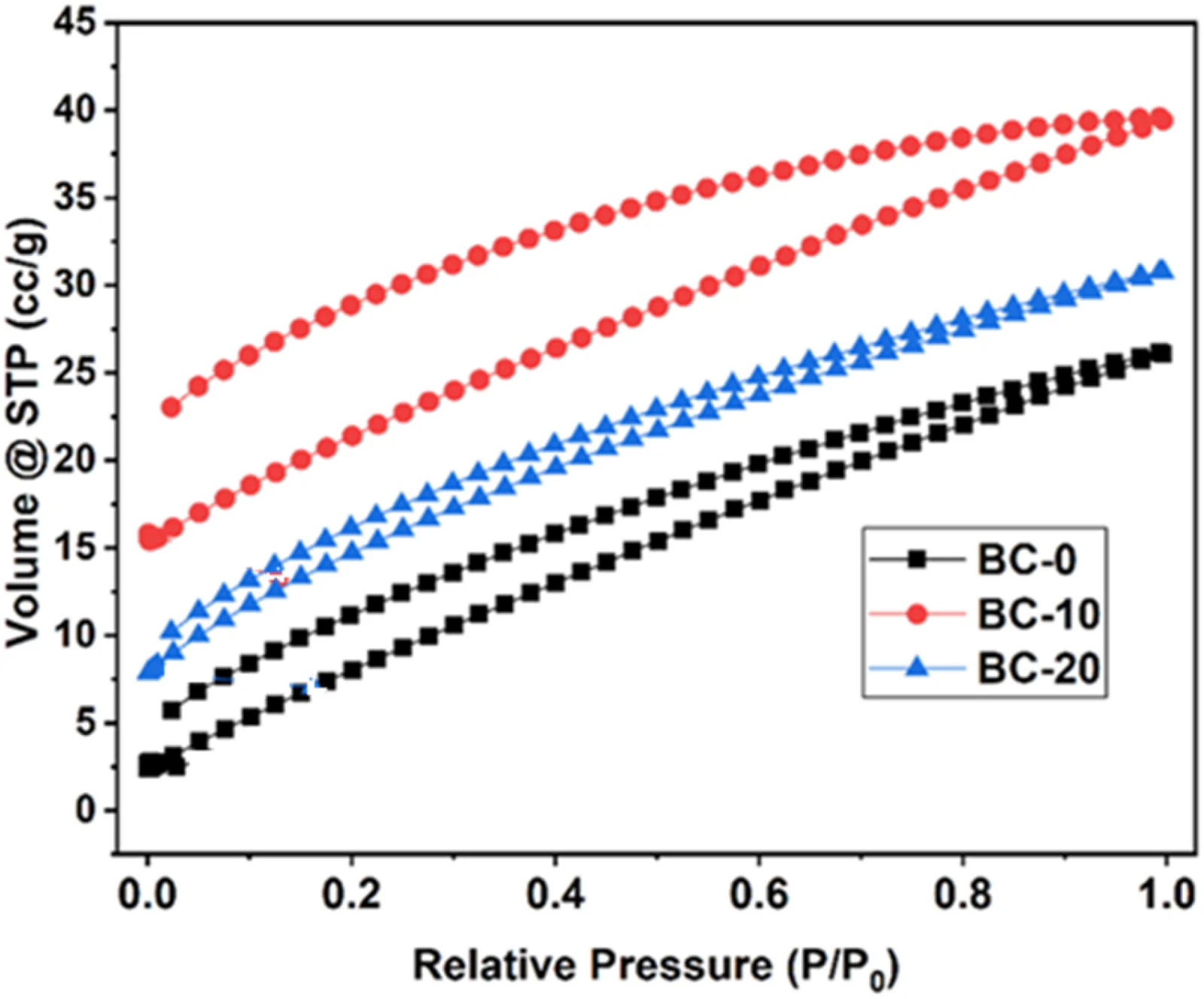
CO_2_ adsorption–desorption of BC0, BC-10 and BC-20.

**Table 5:** Nonlinear fitting parameters and goodness-of-fit statistics. Values reproduced from the supplied analysis; pressure-coordinate units require verification^a^

Sample	Model	Parameters	*R* ^2^	RMSE	SSE	AICc
BC-0	L	2.8243; 0.6704	0.9740	0.0581	0.1789	−297.39
BC-0	F	1.1438; 0.7031	0.9819	0.0485	0.1248	−316.47
BC-10	L	1.5905; 13.4746	0.1930	0.3246	5.3723	−110.53
BC-10	F	1.5226; 0.1713	0.8199	0.1533	1.1992	−187.01
BC-20	L	1.6172; 3.5683	0.7537	0.1726	1.5192	−174.95
BC-20	F	1.2476; 0.2990	0.9111	0.1037	0.5484	−226.92

a L: *q* = *q*_m_*K*^L^*x*/(1 + *K*^L^*x*); parameters *q*_m_, *K*^L^. F: *q* = *K*^f^*x*(1/*n*); parameters *K*^f^, 1/*n*. *x* denotes the pressure coordinate used in fitting. *q*_m_ and RMSE: mmol g^−1^; SSE: (mmol g^−1^)^−2^. RMSE = √(SSE/N). AICc comparisons require the same observations and parameter-count convention; absolute values above are retained from the supplied analysis.

The adsorption branches were fitted using unweighted nonlinear least-squares regression. Freundlich provided higher *R*^2^ and lower RMSE, SSE and AICc values than Langmuir for all three samples (Table 5). The Freundlich *R*^2^ values were 0.9819, 0.8199 and 0.9111 for BC-0, BC-10 and BC-20, respectively, compared with 0.9740, 0.1930 and 0.7537 for the corresponding Langmuir fits. Nevertheless, the remaining deviations for BC-10 indicate that neither two-parameter model adequately captures its complete adsorption response. The statistical preference for Freundlich is consistent with adsorption heterogeneity but does not establish its physical origin or demonstrate interfacial synergism. Compared with BC-0, BC-10 showed an approximately 52% increase in CO_2_ uptake, demonstrating that moderate biochar incorporation effectively optimized pore accessibility and adsorption-site distribution. This enhancement cannot be explained solely by the BET surface area, but rather by the synergistic effects of accessible ultra-micropores, improved particle dispersion, and a greater density of energetically favorable adsorption sites. Similar observations have been reported for activated carbons and aluminum-based MOFs, where CO_2_ adsorption is primarily controlled by pore size distribution and surface chemistry rather than total surface area.^69,72^ Interestingly, the adsorption behavior closely correlates with the CO_2_-TPD results discussed in Section 3.5. The TPD profiles provide a protocol-dependent comparison of desorption response. Selected peak sums refer to peaks centred below 500 °C; they are not absolute adsorption-site densities. The separate feature near 560 °C is excluded from this comparison because decomposition-derived species may contribute. Incomplete calibration and peak-fitting validation limit quantitative interpretation; no direct equivalence with equilibrium uptake is assumed. Polar oxygen-containing groups and Al-containing framework environments may contribute to CO_2_ retention, including possible Lewis acid–base, hydroxyl-associated and electrostatic interactions with the CO_2_ quadrupole. These interactions are proposed rather than directly identified. Literature characterization of MIL-100(Al) acidity^73^ does not establish the identity or abundance of CO_2_-binding sites in the present samples.

Conversely, BC-20 exhibited lower adsorption performance despite retaining a hierarchical pore structure. For an ideal additive mixture, *X*_mix_ = (1 − *w*)*X*_BC-0_ + *wX*_A-BC_, with *w* defined on the final composite-mass basis. This fraction is unknown here. Dilution may contribute to the lower mass-normalized surface area, while pore restriction or altered framework formation are additional possible explanations. Their contributions cannot be separated using nominal precursor fractions or without a composition-matched control. These observations are consistent with previous reports on MOF/carbon composites in which excessive carbon loading diminished the accessibility of framework micropores.^48,62,63,74^ The narrow H4 hysteresis loops observed for all samples indicate the presence of slit-shaped pores originating from the activated carbon matrix and interparticle voids generated during hybrid formation. Mesopores and interparticle voids may provide transport pathways connecting the external surface with narrower adsorption domains. Such pore connectivity would be expected to facilitate molecular access in a hierarchical porous material. However, the present equilibrium isotherms do not establish faster adsorption, lower diffusion resistance, or improved mass-transfer kinetics. Uptake-versus-time measurements and kinetic modelling would be required to evaluate these properties directly.^46,70,74^

An A-BC CO_2_ isotherm and a physical mixture matched to the final composite composition are unavailable. Consequently, the contributions of biochar adsorption, changes in accessible porosity and possible framework–biochar interactions cannot be separated, and an advantage over physical mixing is not established. Independent synthesis-batch replicates and associated uncertainty estimates are also unavailable; the reported differences are descriptive rather than statistically validated. The single-component measurements provide comparative equilibrium screening, while performance under humid mixed-gas conditions and repeated adsorption–desorption cycling remains unverified.

The equilibrium adsorption data were satisfactorily described by both the Langmuir and Freundlich isotherm models. The superior fit of the Freundlich model indicates that CO_2_ adsorption occurs predominantly on energetically heterogeneous surfaces, consistent with the coexistence of MOF-derived adsorption sites and oxygen-containing functional groups on the activated biochar. Overall, the combined CO_2_ adsorption isotherms, textural analysis, and CO_2_-TPD results consistently demonstrate that BC-10 provides the most favourable balance between accessible microporosity and adsorption-site density. Moderate incorporation of activated bamboo-derived biochar preserves MOF-derived ultra-micropores while simultaneously increasing accessible oxygen-containing surface functionalities.^45,48^ Conversely, excessive biochar loading partially blocks the microporous network and reduces the availability of high-energy adsorption sites. Consequently, the overall CO_2_ adsorption performance follows the order BC-10 > BC-20 > BC-0, demonstrating that a 10 wt% biochar loading provides the optimum hierarchical pore architecture for efficient CO_2_ capture.^62,69,72^

### 3.7 Proposed interfacial mechanism for CO_2_-interaction behavior

Fig. 8 illustrates a proposed interfacial mechanism for the formation and CO_2_-interaction behaviour of the MIL-100(Al)/MIL-110(Al)/biochar hybrids, based on the combined results of PXRD, FTIR, SEM, textural, TGA and CO_2_-TPD. The adsorption trends may reflect differences in accessible pore environments^72^ and framework or carbon surface chemistry. Acid sites characterized in MIL-100(Al) provide context for possible polar adsorption environments,^73^ but do not identify the CO_2_-binding sites in these composites. Wider access pathways are plausible in hierarchical porous materials.^75^ These proposed contributions remain non-unique and do not establish an interface-specific mechanism. Hence, the activated bamboo-derived biochar scaffold is proposed to impact the solvothermal assembly of the aluminium trimesate frameworks by offering a heterogeneous carbonaceous surface with oxygenated functional groups. FTIR analysis confirmed the presence of hydroxyl- and carboxyl-containing functionalities on A-BC, and SEM observations revealed that the incorporation of biochar affected the spatial distribution and aggregation behaviour of the MOF crystallites. These findings are consistent with the biochar surface acting as a scaffold to support nucleation during framework growth, which facilitates the more dispersed generation of MIL-100(Al)/MIL-110(Al) crystallites throughout the carbon matrix.^37,40–43^ It is suggested that the adsorption mechanism is due to a combination of micropore filling and heterogeneous surface interaction. The high CO_2_ uptake at very low relative pressure confirms that the initial adsorption process is dominated by the micropore filling in ultra-micropores, where the overlapping adsorption potentials generate strong confinement effects for the CO_2_ molecules. At the same time, coordinatively unsaturated aluminium centres in the MIL framework serve as Lewis acid sites, while hydroxyl, carboxyl and phenolic groups on the activated biochar provide polar adsorption sites that can interact with the quadrupole moment of CO_2_*via* weak Lewis acid–base and dipole–quadrupole interactions.^57–61^ The good fitting of the Freundlich isotherm model is also evidence of the adsorption on heterogeneous energetic surfaces due to the synergistic integration of the aluminium based framework and the activated biochar.

**Fig. 8:**
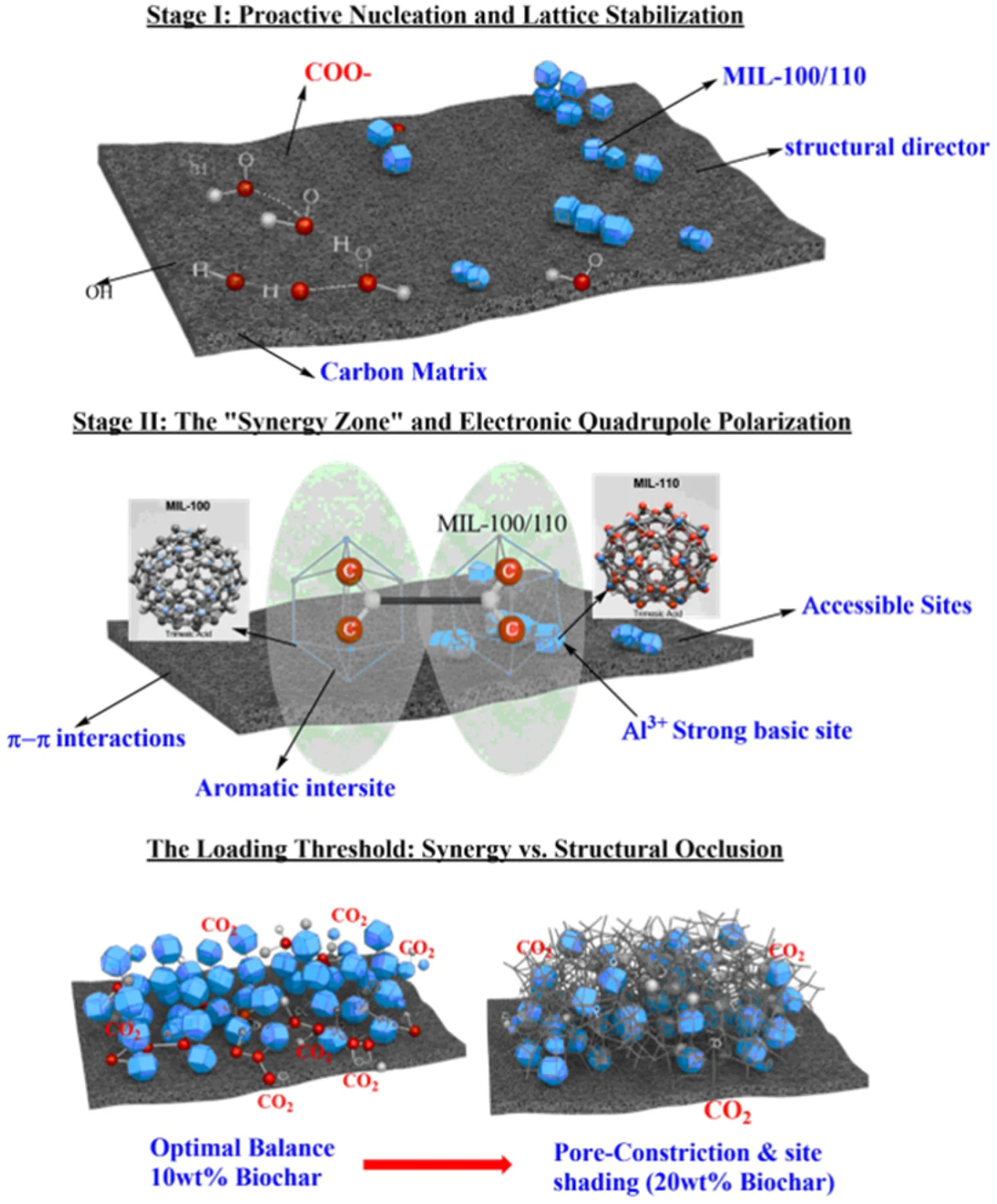
Proposed contributions of pore accessibility, Al-containing environments and oxygenated biochar groups to CO_2_ adsorption in MIL-100(Al)/MIL-110(Al)–biochar hybrids. Molecular interactions and pore restriction remain hypotheses.

The results of CO_2_-TPD and equilibrium adsorption agree with each other in determining the optimum composition of BC-10. The maximum total CO_2_-interaction-site density below 400 °C (6.827 mmol g^−1^) and the highest equilibrium CO_2_-adsorption capacity (39.59 cm^3^ STP per g, 1.77 mmol g^−1^) were observed for the BC-10 sample, suggesting that moderate biochar loading optimises the accessibility of the adsorption sites while preserving the intrinsic porosity of the dual-phase framework. BC-20, in contrast, possessed a lower BET surface area (381.23 m^2^ g^−1^), lower CO_2_-interaction-site density (4.984 mmol g^−1^), and lower equilibrium adsorption capacity (30.77 cm^3^ STP per g, 1.37 mmol g^−1^). These results suggest that over loading biochar partially masks micropores derived from the MOF, restricts the accessibility of the pores and reduces the amount of accessible adsorption sites without majorly changing the primary aluminum–trimesate coordination framework. The proposed mechanism shows that the CO_2_-interaction behaviour of the dual-phase MIL-100(Al)/MIL-110(Al)/biochar hybrids are governed by a biochar-loading-dependent structure–property relationship. A moderate amount of biochar (10 wt%) improves the dispersion of crystallites, retains accessible microporosity and increases the density of heterogeneous adsorption sites, resulting in the highest CO_2_ uptake of the materials investigated. In contrast, the high loading of biochar reduces the pore accessibility and partially masks the adsorption sites from the framework. These results suggest that it is the engineering of the interface rather than the increase of surface area that governs the CO_2_ adsorption performance in the present dual-phase aluminum-based MOF/biochar system.

### 3.8 Comparative discussion

Table 6 compares the CO_2_ adsorption performance of the present dual-phase MIL-100(Al)/MIL-110(Al)–biochar hybrids with representative MOF-based and MOF/carbon adsorbents reported in the literature. Among the synthesized materials, BC-10 exhibited the highest equilibrium CO_2_ adsorption capacity of 1.77 mmol g^−1^ at 298 K and 1 bar, corresponding to approximately 52% and 29% enhancements relative to BC-0 and BC-20, respectively. Notably, this improvement was achieved despite the lower BET and micropore surface areas of BC-10 compared with the pristine dual-phase framework, indicating that CO_2_ uptake is governed not only by total surface area but also by pore accessibility and the distribution of CO_2_-interacting sites. This interpretation is consistent with the CO_2_-TPD results, in which BC-10 showed the highest cumulative weak-to-strong CO_2_-interaction contribution below 400 °C.

**Table 6:** Comparison of the present dual-phase MOF/biochar hybrids with representative MOF/biochar CO_2_ adsorbents

Adsorbent	BET SSA (m^2^ g^−1^)	*T* (K)/*P* (bar)	CO_2_ uptake (mmol g^−1^)	Adsorption model	Main feature	Ref.
BC-0	1149	298/1	1.16	Freundlich	PXRD-assigned phase coexistence; reference material	This work
BC-10	497	298/1	1.77	Freundlich	Highest uptake among the three tested formulations	This work
BC-20	381	298/1	1.37	Freundlich	Lower accessible texture and uptake than BC-10	This work
MIL-100(Fe,Al)/biochar	∼1450	298/1	3.21	Dual-site Langmuir	Dual open-metal adsorption sites	76
Zn–Cu-BTC/Carbon foam	∼1380	308/1	3.97	Langmuir–Freundlich	Enhanced CO_2_/N_2_ selectivity	77
CoNi-MOF-74/biochar	∼1100	298/1	7.55	Hill	High CO_2_ affinity from dual metal centers	78
CdMg-MOF-74/biochar	—	298/1	4.90	Dual-site Langmuir	Amine-functionalized adsorption sites	79

The comparison further highlights that adsorption performance depends strongly on framework composition, pore architecture, activation strategy, adsorption temperature, pressure, and surface chemistry. Several reported MOF-based adsorbents achieve higher CO_2_ capacities through high-surface-area frameworks, open metal sites, bimetallic nodes, or post-synthetic functionalization. In contrast, the present work demonstrates a simple biochar-assisted interfacial-engineering strategy for tuning adsorption-site accessibility in aluminum-based dual-phase MOF hybrids. Therefore, the main contribution of this study lies not in achieving the highest absolute CO_2_ uptake, but in establishing how controlled biochar incorporation regulates framework dispersion, accessible microporosity, heterogeneous adsorption-site distribution, and equilibrium CO_2_ adsorption behavior.

Overall, the principal contribution of this work is the establishment of a biochar-loading-dependent structure–property relationship in dual-phase aluminum trimesate–biochar hybrids. Moderate biochar incorporation suppresses particle agglomeration, promotes a more dispersed hybrid morphology, preserves accessible microporosity, and introduces additional oxygen-containing surface functionalities. These combined effects generate energetically heterogeneous adsorption environments, as reflected by the Freundlich model fitting and enhanced CO_2_-TPD response of BC-10. In contrast, excessive biochar loading partially restricts pore accessibility and masks framework-derived adsorption sites, resulting in lower CO_2_ uptake. These findings provide design guidance for biomass-derived MOF hybrid adsorbents in which interfacial architecture and adsorption-site accessibility are optimized rather than relying solely on increasing surface area.

## 4. Conclusion

Activated bamboo-derived biochar was incorporated during the *in situ* synthesis of aluminum trimesate–biochar hybrids to examine how nominal biochar addition affects morphology, accessible porosity and equilibrium CO_2_ adsorption. PXRD features are consistent with the coexistence of phases assigned to MIL-100(Al) and MIL-110(Al), while microscopy reveals composition-dependent morphological differences. These observations do not establish a crystallization-directing role for biochar or a crystallographically integrated dual-phase interface. BC-10 and BC-20 denote precursor-based synthesis formulations; their final biochar mass fractions remain unquantified.

BC-10 was the best-performing composition among the investigated samples, exhibiting an equilibrium CO_2_ uptake of 39.59 cm^3^ STP per g (approximately 1.77 mmol g^−1^), approximately 52% higher than BC-0, despite its lower BET surface area of 496.79 m^2^ g^−1^. This comparison indicates that BET surface area alone does not explain the observed uptake trend. The lower uptake of BC-20 may reflect dilution, changes in framework formation or reduced pore accessibility; these contributions cannot be separated from the available measurements. Freundlich fitting provides a better statistical description than Langmuir fitting, although neither model fully represents BC-10. This preference is consistent with a heterogeneous adsorption response but does not identify its molecular origin. CO_2_-TPD is interpreted as a protocol-dependent desorption response rather than an absolute adsorption-site density, and TGA characterizes thermal decomposition under N_2_ rather than operational stability.

The study identifies descriptive composition-dependent adsorption trends rather than a statistically validated structure–property relationship or demonstrated interfacial synergism. Without biochar-only CO_2_ adsorption, a composition-matched physical mixture and independent replicates, the contributions of the individual components and the reproducibility of the observed enhancement remain unresolved. Proposed molecular interactions and transport benefits therefore remain hypotheses. Additional compositions and multitemperature, kinetic, humid mixed-gas, cycling and breakthrough measurements are required to establish the loading optimum and assess application-relevant performance.

## Author contributions

The manuscript was written through contributions of all authors. All authors have given approval to the final version of the manuscript.

## Conflicts of interest

There are no conflicts to declare.

## Data Availability

The data supporting the findings of this study are included within the article.
